# A novel regulatory network among *LncRpa*, *CircRar1*, *MiR*-*671* and apoptotic genes promotes lead-induced neuronal cell apoptosis

**DOI:** 10.1007/s00204-016-1837-1

**Published:** 2016-09-07

**Authors:** Aruo Nan, Lijian Chen, Nan Zhang, Zhenzhong Liu, Ti Yang, Zhishan Wang, Chengfeng Yang, Yiguo Jiang

**Affiliations:** 1 State Key Laboratory of Respiratory Disease, Institute for Chemical Carcinogenesis, Guangzhou Medical University, Xinzao, Panyu District, Guangzhou, 511436 China; 2 Department of Physiology, Michigan State University, East Lansing, MI 48824 USA; 3 Institute for Integrative Toxicology, Michigan State University, East Lansing, MI 48824 USA

**Keywords:** CircRNA, LncRNA, MiRNA, Cell apoptosis, Lead, Neurotoxicity

## Abstract

Lead is a metal that has toxic effects on the developing nervous system. However, the mechanisms underlying lead-induced neurotoxicity are not well understood. Non-coding RNAs (ncRNAs) play an important role in epigenetic regulation, but few studies have examined the function of ncRNAs in lead-induced neurotoxicity. We addressed this in the present study by evaluating the functions of a long non-coding RNA (named *lncRpa*) and a circular RNA (named *circRar1*) in a mouse model of lead-induced neurotoxicity. High-throughput RNA sequencing showed that both *lncRpa* and *circRar1* promoted neuronal apoptosis. We also found that *lncRpa* and *circRar1* induced the upregulation of apoptosis-associated factors caspase8 and p38 at the mRNA and protein levels via modulation of their common target microRNA *miR*-*671*. This is the first report of a regulatory interaction among a lncRNA, circRNA, and miRNA mediating neuronal apoptosis in response to lead toxicity.

**Electronic supplementary material:**

The online version of this article (doi:10.1007/s00204-016-1837-1) contains supplementary material, which is available to authorized users.

## Introduction

Lead is an industrial and environmental pollutant that can cause pathological changes to multiple organ systems, including the nervous system. Lead has irreversible neurotoxic effects on the developing brain (Wang et al. 2008). However, the molecular mechanisms of lead-induced neurotoxicity remain unclear. There have been few studies investigating the role of non-coding RNAs (ncRNAs) in this process, although they have been shown to play important roles in many biological processes, including transcriptional regulation, DNA replication, RNA processing, mRNA stability and translation, and protein degradation and transport (Storz 2002).

Long non-coding RNAs (lncRNAs) and circular RNAs (circRNAs) have been the focus of many recent studies on ncRNA function. LncRNAs are 200 nucleotides in length and are distributed in the nucleus or cytoplasm (Maruyama and Suzuki 2012; Okazaki et al. 2002). LncRNAs regulate gene expression at the transcriptional, post-transcriptional, and epigenetic levels and have been implicated in species evolution, embryonic development, metabolism and disease (Mercer et al. 2009; Taft et al. 2010). On the other hand, knowledge of circRNA functions is limited. These molecules were originally identified in an RNA virus (Wilusz and Sharp 2013); they are widely present in mammalian cells and have been linked to the regulation of gene expression (Zhang et al. 2014), alternative splicing (Lasda and Parker 2014), and translation through interaction with RNA-binding proteins (Zhang et al. 2013). CircRNAs have been shown to bind microRNAs (miRNAs) as sponges, thereby indirectly regulating target gene expression (Hansen et al. 2013a; Wilusz and Sharp 2013). There have been no reports to date of interactions between lncRNAs and circRNAs.

The present study investigated the functions of lncRNAs and circRNAs in lead-induced neurotoxicity. We first carried out an RNA screen in a mouse model. A quantitative real-time polymerase chain reaction (qRT-PCR) analysis revealed that the pro-apoptotic lncRNA (named *lncRpa)* and the apoptosis-related circRNA (named *circRar1)* were upregulated in the hippocampus and cerebral cortex of mice with lead-induced neurotoxicity. A similar upregulation of *lncRpa* and *circRar1* was observed in N2a cells treated with lead acetate (PbAc). We found that *lncRpa* and *circRar1* acted via the common target *miR*-*671* to promote neuronal apoptosis. These findings highlight the regulatory roles of lncRNAs and circRNAs in lead-induced neurotoxicity and provide the first evidence of these ncRNAs modulating a cellular process by jointly targeting a specific miRNA.

## Materials and methods

### Lead-induced neurotoxicity models

The mouse model of lead-induced neurotoxicity has been previously described, and the animal studies were approved by the Animal Care and Use Committee of Guangzhou Medical University (Nan et al. 2016). We also used mouse neuroblastoma N2a cells exposed to PbAc at a concentration of 0.1 µM for 48 h as an in vitro model of lead-induced neurotoxicity (Nan et al. 2016).

### RNA extraction

RNA was obtained from brain tissue (cerebellum, pons, medulla oblongata, hippocampus, and cerebral cortex) of mice with lead-induced neurotoxicity (2 and 5 weeks of exposure to PbAc) and control mice. Trizol reagent (Invitrogen, Carlsbad, CA, USA) was used according to the manufacturer’s instructions to extract total RNA from tissues and cells. For quantitation of circRNAs, RNase R (Epicentre, Madison, WI, USA) was added to degrade linear RNAs. RNA quality and concentration were measured with a NanoDrop1000 spectrophotometer (NanoDrop Technologies, Wilmington, DE, USA).

### High-throughput RNA sequencing

The HiSeq 2000 sequencing platform (Illumina, San Diego, CA, USA) was used for high-throughput RNA sequencing. The protocol involved removal of rRNA, followed by synthesis of double-stranded cDNA and end repair. After linking sequencing adaptors and selecting fragments, the second strand of cDNA was degraded and the remaining strand was enriched by PCR. The quality of the library was confirmed by sequencing. A bioinformatic analysis of the raw sequencing data was carried out. Differentially expressed ncRNAs were searched in the NCBI database (http://www.ncbi.nlm.nih.gov/) to determine their genome loci.

### qRT-PCR

The Goscript Reverse Transcription System (Promega, Madison, WI, USA) was used to reverse transcribe lncRNAs, circRNAs, and mRNAs to cDNA. Go Taq qPCR Master Mix (Promega) was used for qRT-PCR. All-in-one miRNA qRT-PCR Detection kit (Genecopoeia, Rockville, MD, USA) was used to reverse transcribe and amplify miRNAs. *Glyceraldehyde 3*-*phosphate dehydrogenase (GAPDH)* was used as an internal control for the relative quantitation of lncRNAs, circRNAs, and mRNAs, whereas *U6* was used for miRNAs. The detection of internal control gene *GAPDH* would be affected after treating with RNase R; we divided the same RNA sample into two uniform parts when performing the qRT-PCR experiment. One part was treated with RNase R for delinearization; this part was for the further detection of circRNA. The other part was treated with RNase R-free water for finally detecting *GAPDH* gene. The primer sequences are shown in Supplementary Table 3. The 2^−ΔΔCt^ method was used to determine relative expression levels.

### RNA interference and overexpression

LncRNA and circRNA expression was suppressed by siRNA-mediated knockdown. Three different siRNAs were designed and tested for both *lncRpa* and *circRar1*. Overexpression vectors for *lncRpa* and *circRar1* were also constructed (BersinBio, Guangzhou, China). CircRNA upstream intron cyclization component (526 bp), circRNA (462 bp) and circRNA downstream intron cyclization component (804 bp) were included in circRNA expression area. BamHIand Hind III were jointly connected to expression vector pcDNA 3.1+ through double enzyme connection. Overexpression and siRNA sequences are shown in Supplementary Table 1. A specific inhibitor and mimic (RiboBio, Guangzhou, China) were used to inhibit or induce *miR*-*671* expression, respectively. Cells were transfected with plasmid using EndoFectin Lenti reagent (Genecopoeia). RiboFECT CP Transfection kit (166T) (RiboBio) was used for the *miR*-*671* inhibitor and mimic.

### Detection of cell apoptosis by FCM

The Annexin V-fluorescein isothiocyanate (FITC) apoptosis assay kit (KeyGen Biotech, Nanjing, China) was used according to the manufacturer’s instructions to detect apoptotic cells 48 h after transfection and PbAc treatment. Briefly, 5 × 10^5^ cells were collected and resuspended in 100 μl 1 × binding buffer. Five microliters Annexin V-FITC and 5 μl propidium iodide staining solution were added to the cells, followed by incubation at room temperature (shielded from light) for 10 min. Four hundred microliters 1 × binding buffer was added to the reaction, and cells were analyzed by FCM (BD Biosciences, Franklin Lakes, NJ, USA) within 1 h.

### Detection of cell apoptosis by TUNEL assay

A TUNEL kit (Roche Diagnostics, Indianapolis, IN, USA) was used to detect apoptotic cells. Cells were cultured on Lab-Tek chambered slides (Thermo Fisher Scientific, Waltham, MA, USA). Following treatment, the samples were washed twice with phosphate-buffered saline (PBS) and fixed with 4 % paraformaldehyde at room temperature for 20 min, followed by two washes with PBS. Proteinase K (20 μg/ml; Sangon Biotech, Shanghai, China) was added, and the slides were covered with a film and incubated at 37 °C for 20 min and then washed twice with PBS. The TUNEL reaction mixture (enzyme and labeling solutions at a 1:9 ratio) was added to the slides, which were covered with film and incubated at 37 °C for 60 min. After three washes with PBS, converter-peroxidase was added at 37 °C for 30 min; after three more washes with PBS, diaminobenzidine reagent (Roche Diagnostics) was added at room temperature for 10 min. The samples were washed three times with PBS and counterstained with hematoxylin for 10 s and then washed with running water. After dehydration in a graded series of alcohol, the samples were dried and mounted with neutral balsam. Nuclei with yellowish brown staining were positive (apoptotic), and hematoxylin-counterstained intact nuclei appeared blue under a light microscope.

### CCK-8 cell viability assay

The CCK-8 assay kit (Beyotime Institute of Biotechnology, Shanghai, China) was used to assess cell viability. Cells were harvested in logarithmic phase, and 100 μl of the suspension (~2000 cells) were seeded in each well of a 96-well plate and incubated overnight at 37 °C and 5 % CO_2_. EndoFectin Lenti reagent, plasmids, siRNAs, and serum-free Dulbecco’s Modified Eagle’s Medium (DMEM; HyClone, Logan, UT, USA) equilibrated to 15–25 °C were added to the wells followed by incubation at room temperature for 10–25 min. The medium was changed after 6 h, and 0.1 μM PbAc solution was added for 48 h. Ten microliters CCK-8 solution was added for 1–4 h, and the absorbance at 450 nm was measured using a microplate reader.

### Western blotting

Total protein was extracted using a commercial kit (KeyGen Biotech). Protein samples (4–8 μg/μl) were mixed with a 4:1 ratio of 5 × loading buffer and β-mercaptoethanol and stored at −80 °C until use. Proteins (40–60 μg per well) were separated by sodium dodecyl sulfate polyacrylamide gel electrophoresis (100–120 V). A protein marker with a molecular weight range of 16–220 kDa was used as reference. The proteins were transferred to a polyvinylidene difluoride membrane (Merck Millipore, Billerica, MA, USA) at 200 mA using a wet membrane-transfer device (Bio-Rad, Hercules, CA, USA). The membrane was washed with Tris-buffered saline containing 0.1 % Tween-20 (TBST) for 1–2 min and then blocked at room temperature for 60 min with TBST containing 5 % non-fat milk powder. After overnight incubation at 4 °C with primary antibodies, the membrane was washed with TBST three times for 15 min. The membrane was then incubated at room temperature for 60 min with secondary antibody and washed three times with TBST for 15 min each. Protein bands were visualized using the BeyoECLPlus chemiluminescence reagent (Beyotime Institute of Biotechnology) followed by exposure to X-ray film. Primary antibodies against the following proteins were used in this study: caspase3 (Cell Signaling Technology, Danvers, MA, USA), caspase9 (Epitomics, Burlingame, CA, USA), Akt2 (Cell Signaling Technology), caspase 8 (Proteintech, Rosemont, IL, USA), and p38 (Cell Signaling Technology). The secondary antibody was horseradish peroxidase-conjugated IgG (Boster Bio, Pleasanton, CA, USA).

## FISH

Cells grown on coverslips were fixed with 4 % paraformaldehyde at room temperature for 15 min, washed twice with 0.1 % diethylpyrocarbonate solution and treated with 0.5 % Triton X-100 at room temperature for 5 min. The samples were dehydrated in a graded series of alcohol and air-dried. After adding probe hybridization solution, the samples were mounted, denatured at 73 °C for 3 min, and hybridized in a humid and dark environment at 37 °C for 12–16 h with Cy3-labeled miRNA probe, 6-carboxyfluorescein-labeled circRNA probe, and Cy5-labeled lncRNA probe (BersinBio). The samples were washed three times with a pre-heated (43 °C) solution consisting of 50 % formamide and 2× saline sodium citrate (SSC), and then washed twice with 2× SSC (37 °C). After counterstaining with 4′, 6-diamidino-2-phenylindole, the samples were mounted with fluorescence mounting medium and imaged with a microscope.

### Dual luciferase reporter gene assay

Cells were seeded and incubated for 24 h. At 80–90 % confluence, the cells were transfected with firefly and *Renilla* luciferase plasmids. After washing with PBS, passive lysis buffer (PLB) was added and cells were incubated at room temperature for 15 min, with a micro-oscillator used to lyse the cells. Lysates were centrifuged at 10,000 rpm for 5 min at 4 °C. The supernatants were removed, and 20 μl sample were transferred to a 96-well plate and mixed with 100 μl Dual-Glo Luciferase Assay System (Promega), with cell lysis buffer used as the control. The relative light units were measured before and after adding 100 μl Stop & Glo reagent.

### RNA antisense purification (RAP)

The RAP kit (BersinBio) was used for this experiment. RAP employs specific biotinylated probes that hybridize to target RNAs (mRNAs or miRNAs); these can then be pulled down, reverse transcribed to cDNA, and identified by qRT-PCR or sequencing. A total of 10^7^ cells were washed with PBS and cross-linked by ultraviolet irradiation at 254 nm (0.15 J cm^−2^). Cells were lysed with 1 ml lysis buffer and fully homogenized with a 0.4 mm syringe. Two different 45-bp biotinylated antisense probes (0.2 nmol) were added to the lncRNA-RAP system and one 50-bp biotinylated antisense probe (0.2 nmol) targeting the adaptor sequence was added to the circRNA-RAP system. The probes were denatured at 65 °C for 10 min and hybrided at room temperature for 2 h before adding 200 µl streptavidin-coated magnetic beads. Non-specifically bound RNAs were removed by washing, and Trizol reagent was used to recover miRNAs specifically interacting with ncRNAs. PCR and qRT-PCR were used to analyze binding strength after reverse transcribing the miRNAs. The probes are shown in Supplementary Table 4.

### Statistical analysis

Data are presented as mean ± SD. All experiments were performed at least three times, and western blotting, TUNEL, FCM, and FISH results are representative of three independent experiments. The unpaired t test was used for statistical analyses. * represents statistically significant difference (*p* < 0.05). ** represents highly statistically significant difference (*p* < 0.01). Data were analyzed using SPSS v.19.0 software (IBM, Armonk, NY, USA).

## Results

### Identification of lncRNAs and circRNAs differentially expressed in lead-induced neurotoxicity

**Fig. 1:**
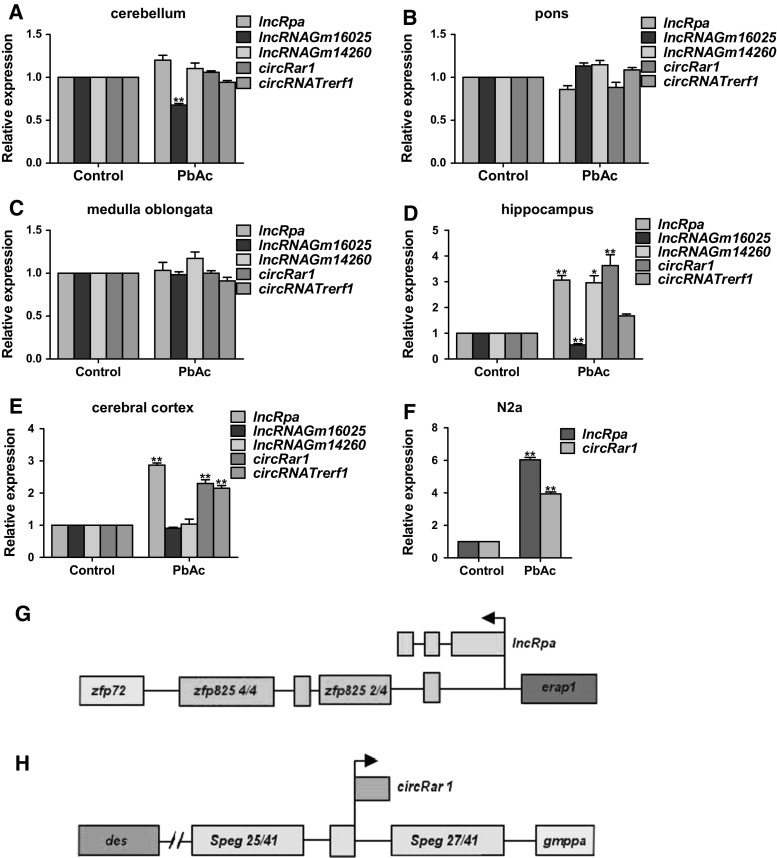
Identification of differentially expressed ncRNAs and their genomic loci. **a**–**e** Relative expression of five genes differentially expressed in the **a** cerebellum, **b** pons, **c** medulla oblongata, **d** hippocampus, and **e** cerebral cortex of mice with lead-induced neurotoxicity, as detected by qRT-PCR. Tissues from mice not treated with PbAc were used as a control. **f** Relative expression of *lncRpa* and *circRar1* in a cellular model of lead-induced neurotoxicity. Cells not treated with PbAc were used as a control. **g** *LncRpa* gene locus map. The lncRNA contains introns and the Zfp825 gene promoter. Different exon regions are represented by zfp72, zfp825 4/4, zfp825 2/4, and erap1. **h** *CircRar1* gene locus map. The circRNA is located in an intron; des, Speg 25/41, Speg 27/41, and gmppa represent different exon regions

A mouse model of lead-induced neurotoxicity was established by PbAc exposure (Nan et al. 2016), and high-throughput RNA sequencing was carried out using the brain tissues of these mice. Three lncRNAs and two circRNAs showing more significant differences in expression between lead-injured mice and controls—including lncRNA *TCONS00001596* (named *lncRpa*), lncRNA *Gm16025* (ENSMUST00000161282), lncRNA *Gm14260* (ENSMUST00000125121), and the circRNAs (located at chr1_75418457_75418970_+, named *circRar1*) and *Trerf1* (located at chr17_47315500_47316549_ +)—were selected for further analysis. The expression of these five molecules in the cerebellum, pons, medulla oblongata, hippocampus, and cerebral cortex was evaluated by qRT-PCR (Fig. 1a–e). The expression of *Gm16025* was downregulated in the cerebellum, while that of the other four RNAs was unaltered (Fig. 1a). None of the RNAs were differentially expressed in the pons and medulla oblongata between injured and control animals (Fig. 1b, c). *LncRpa*, *Gm14260*, and *circRar1* levels were markedly upregulated in the hippocampus, while *Gm16025* was downregulated and circRNA *Trerf1* level showed no change upon injury (Fig. 1d). In the cerebral cortex, *lncRpa*, *circRar1*, and *Trerf1* were upregulated while no changes in *Gm16025* or *Gm14260* were observed (Fig. 1e). *LncRpa* and *circRar1* expression was also upregulated in lead-treated N2a mouse nerve cells(Nan et al. 2016), with higher levels observed for the former (Fig. 1f). The genomic loci of *lncRpa* and *circRar1* were determined (Fig. 1g, h).

### *LncRpa* and *circRar1* promote apoptosis in lead-induced neurotoxicity

**Fig. 2:**
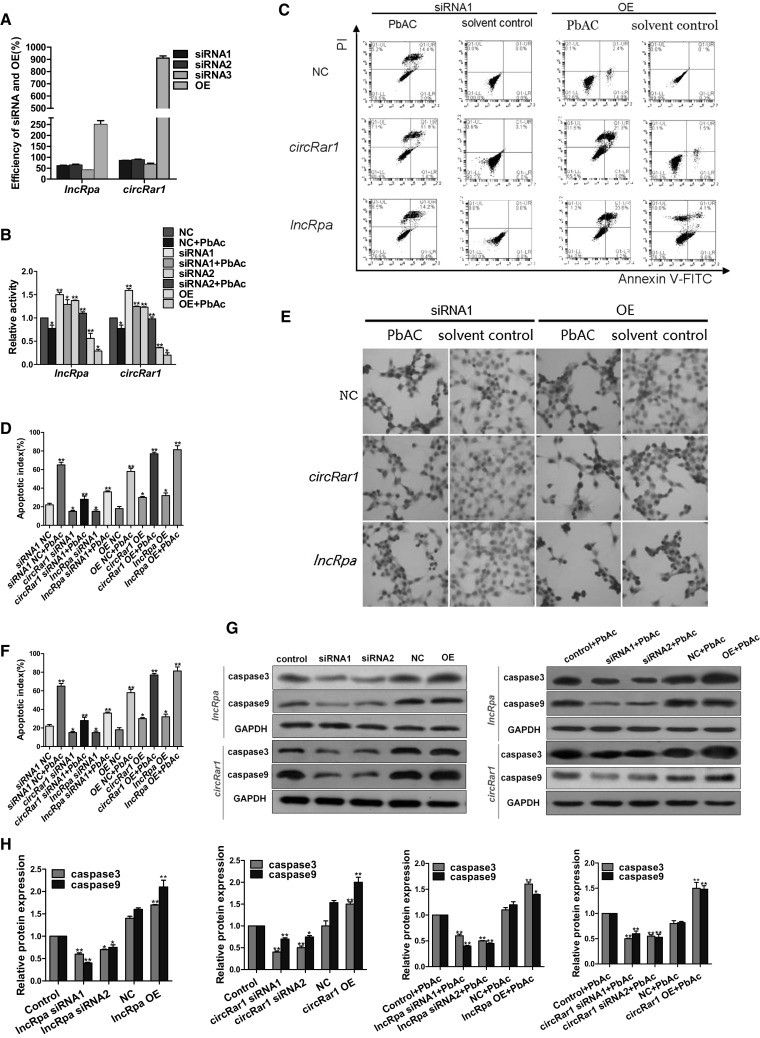
Effects of *lncRpa* and *circRar1* on lead-induced neuronal apoptosis. **a** *LncRpa* and *circRar1* were knocked down by siRNA or overexpressed (OE) in N2a cells; expression levels were detected by qRT-PCR. Overexpression efficiencies of *lncRpa* and *circRar1* were >200 %. **b** Viability of lead-treated N2a cells, as detected by CCK-8. **c** Apoptosis in lead-treated N2a cells, as determined by FCM using annexin V-FITC/PI. LR, early-stage apoptotic cells; UL, necrotic cells; UR, late-stage apoptotic and necrotic cells; LL, living cells. **d** Quantitative analysis of apoptosis rate, calculated as the sum of UR % and LR %. **e** Detection of apoptosis at the chromosome level by the TUNEL assay. Brown nuclei are positive; intact nuclei stained with hematoxylin are blue. **f** Apoptotic index based on TUNEL. Five high-power fields (400 ×) of positive cells were counted and are shown as the percentage of all positive cells (*n* = 500). **g** Caspase9 and caspase3 expression in lead-treated N2a cells, as determined by western blotting. Control and NC represent transfection reagent and empty vector control groups, respectively. **h** Quantitative analysis of results shown in panel (**g**)

We studied *lncRpa* and *circRar1* functions by their overexpression and knockdown in N2a cells. Three short interfering RNAs (siRNAs) as well as overexpression constructs (Supplementary Table 1) were designed for each of *lncRpa* and *circRar1*. The efficiency of knockdown and overexpression was evaluated by qRT-PCR. SiRNA1 and siRNA 2 were more efficient at suppressing *lncRpa* or *circRar1* expression than siRNA 3 (Fig. 2a), and were used in subsequent experiments. Both *lncRpa* and *circRar1* overexpression constructs resulted in higher levels of these two ncRNAs in N2a cells (Fig. 2a). The viability of cells with knockdown or overexpression of *lncRpa* or *circRar1* was evaluated before and after PbAc exposure with Cell Counting Kit-8 (CCK-8)(Fig. 2b), while cell apoptosis was assessed by flow cytometry (FCM) (Fig. 2c, d), terminal deoxynucleotidyl transferase dUTP nick end labeling (TUNEL) (Fig. 2e, f), and detection of caspase9 and caspase3 expression by western blotting (Fig. 2g, h). The rate of apoptosis was higher in the negative control siRNA (NC) + PbAc group than in untreated cells, an effect that was mitigated by *lncRpa* and *circRar1* knockdown prior to PbAc treatment. On the other hand, *lncRpa* and *circRar1* overexpression increased apoptosis in PbAc-treated cells relative to those overexpressing an empty vector as NC. These results suggest that *lncRpa* and *circRar1* promote apoptosis in lead-induced neurotoxicity.

### *LncRpa* and *circRar1* interact directly with *miR*-*671*

**Fig. 3:**
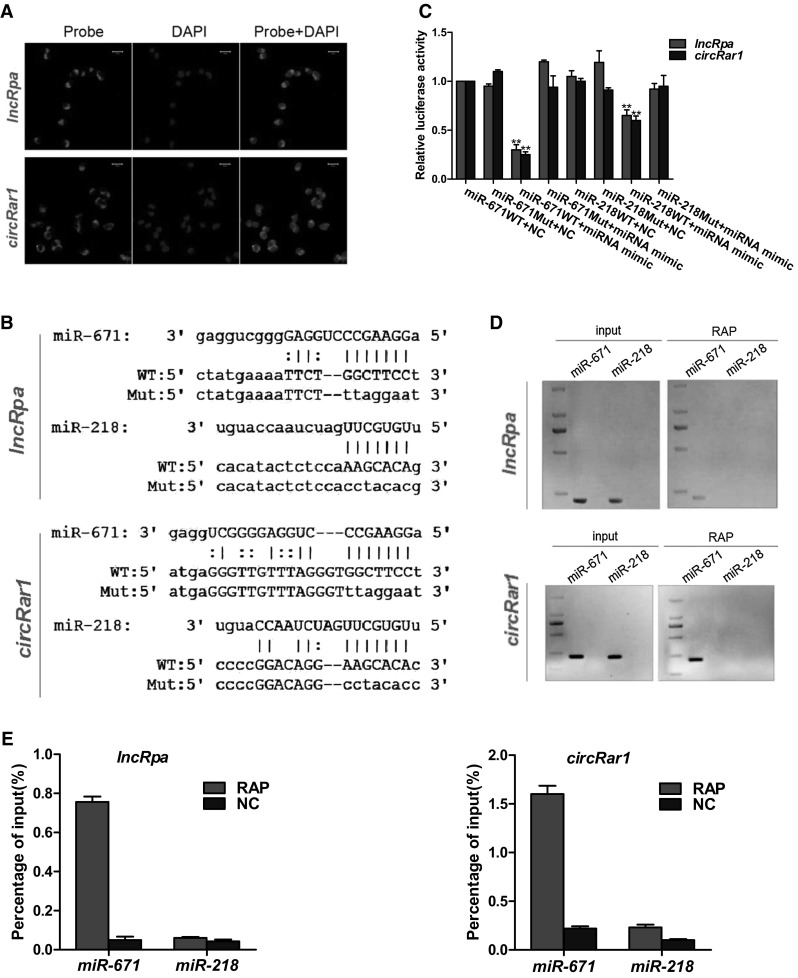
Identification of *lncRpa* and *circRar1* target miRNAs. **a** Detection of *lncRpa* and *circRar1* by FISH. Green represents FISH probes of *lncRpa* and *circRar1*. Nuclei are counterstained with DAPI (*blue*). **b** Alignment of *lncRpa* and *circRar1* and the seed sequences of *miR*-*671* and *miR*-*218*. WT, wild-type sequence; Mut, sequence mutated in the dual luciferase reporter gene assay. **c** Dual luciferase assay. WT, wild-type vector; Mut, mutated vector; NC, blank control; miRNA mimic, *miR*-*671* overexpression. **d** Identification of target miRNAs by RAP. No RAP probes were used for the input control. **e** Percentage of purified miRNAs relative to the input group, as detected by qRT-PCR. NC represents control only with beads (color figure online)

To clarify the mechanism underlying the stimulatory effects of *lncRpa* and *circRar1* in lead-induced apoptosis, we used fluorescence in situ hybridization (FISH) (Supplementary Table 2) to visualize the cellular distribution of these two molecules (Fig. 3a). *LncRpa* and *circRar1* were both expressed in the cytoplasm, suggesting that they function via post-transcriptional mechanisms. We identified miRNAs that were predicted to interact with *lncRpa* and *circRar1* using miRanda, Target Scan, and RegRNA (Padmashree and Swamy 2015; Hsu et al. 2007; Huang et al. 2006). Alignment of these miRNAs with seed sequences revealed that *miR*-*671* and *miR*-*218* interacted with both *lncRpa* and *circRar1* (Fig. 3b). The dual luciferase reporter assay revealed that these miRNAs interact directly with *lncRpa* and *circRar1*, with *miR*-*671* showing stronger binding to *lncRpa* and *circRar1* (Fig. 3c). RNA antisense purification (RAP) and transcriptome sequencing analyses indicated that only *miR*-*671* was a shared target of *lncRpa* and *circRar1.* The miRNAs identified by RAP were analyzed by qRT-PCR and gel electrophoresis. Compared to the input group with no RAP probe, *miR*-*671* was present, whereas *miR*-*218* was absent in the RAP group (Fig. 3d). Moreover, *lncRpa* and *circRar1* bound *miR*-*671* in the RAP but not the control group (Fig. 3e). These results indicate that *lncRpa* and *circRar1* interact directly and specifically with *miR*-*671*.

### *LncRpa* and *circRar1* regulate *miR*-*671* expression

**Fig. 4:**
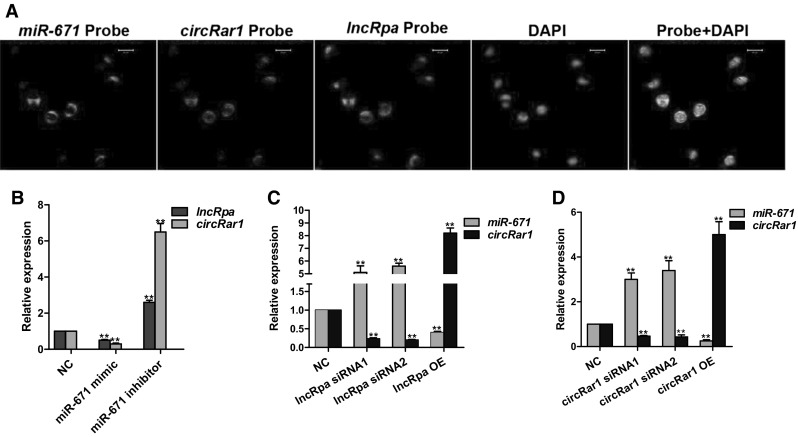
Interaction of *lncRpa*, *circRar1* and *miR*-*671*. **a** Co-localization of *lncRpa* (*magenta*), *circRar1* (*green*), and *miR*-*671* (*red*) in N2a cells. Nuclei were counterstained with DAPI (*blue*). **b**–**d** Overexpression (OE; using a mimic for *miR*-*671* and overexpression vectors for *lncRpa* and *circRar1*) and siRNA-mediated knockdown of **b** *miR*-*671*, **c** *lncRpa*, and **d** *circRar1* (color figure online)

The cellular localization of *lncRpa*, *circRar1,* and *miR*-*671* was evaluated by FISH using the probes (Supplementary Table 2). All three molecules were co-expressed in the cytoplasm of N2a cells in the same pattern (Fig. 4a). In order to investigate the interaction between the three molecules, *miR*-*671* was overexpressed or knocked down and *lncRpa* and *circRar1* levels were evaluated by qRT-PCR. *MiR*-*671* suppression resulted in the upregulation of *lncRpa* and *circRar1,* whereas *miR*-*671* overexpression inhibited their expression (Fig. 4b). We also assessed the interaction between the three molecules by altering the expression levels of *lncRpa* or *circRar1* and observing the effect on the expression of the other two molecules. We found that *miR*-*671* expression was upregulated whereas that of *circRar1* was downregulated upon *lncRpa* knockdown. On the other hand, *lncRpa* overexpression resulted in a decrease in *miR*-*671* and increase in *circRar1* levels (Fig. 4c). Similarly, *circRar1* suppression increased *miR*-*671* and decreased *lncRpa* expression whereas *circRar1* overexpression had the opposite effect (Fig. 4d). These results suggest negative regulation between *lncRpa*/*circRar1* and *miR*-*671* and a positive regulatory relationship between *lncRpa* and *circRar1*.

### *MiR*-*671* inhibits apoptosis

**Fig. 5:**
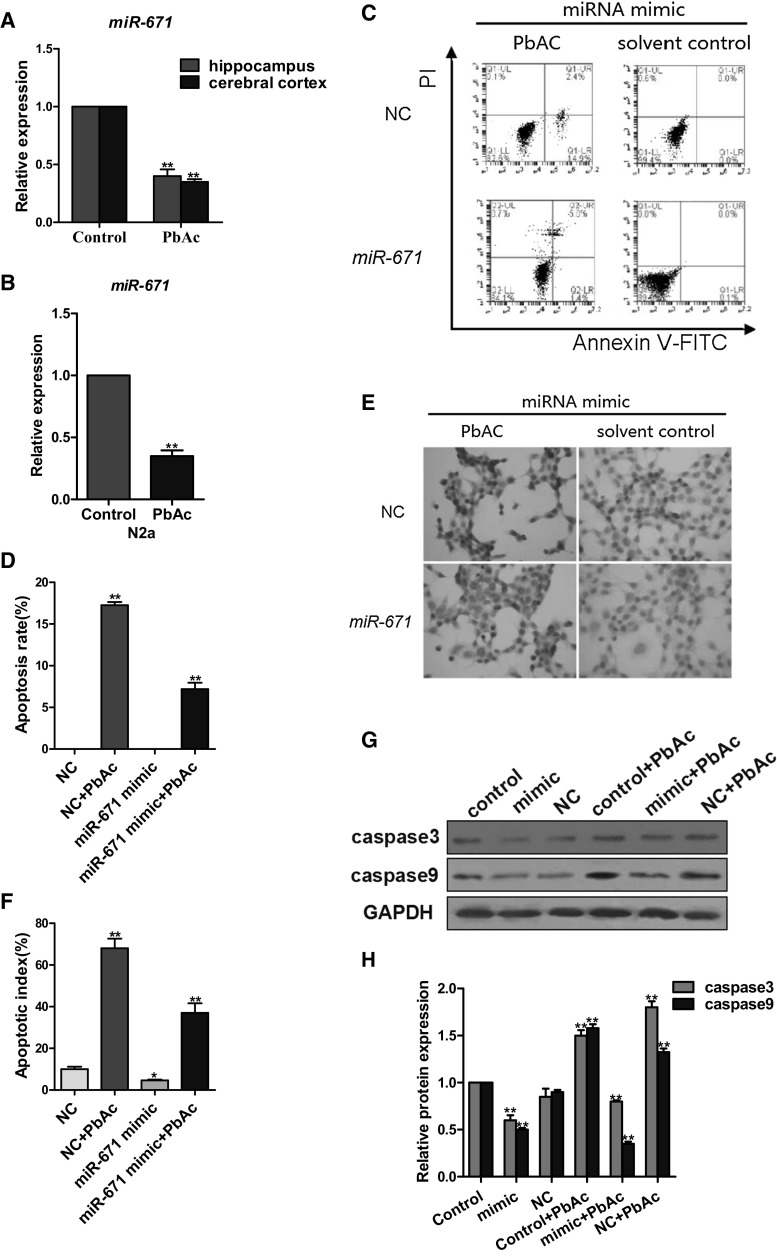
Analysis of *miR*-*671* function. **a**, **b** Downregulation of *miR*-*671* expression in the hippocampus and cerebral cortex in a mouse model of lead-induced neurotoxicity (**a**) and in PbAc-treated N2a cells (**b**). Controls were tissue from mice and N2a cells that were not treated with PbAc. **c** Apoptosis of N2a cells expressing *miR*-*671* mimic, as determined by FCM using annexin V-FITC/PI. NC, transfection reagent negative control group. **d** Quantitative analysis of apoptosis rate, calculated as the sum of UR % and LR %. **e** Apoptosis of N2a cells overexpressing *miR*-*671*, as detected with TUNEL. **f** Apoptotic index calculated from results in panel (**e**). **g** Caspase9 and caspase3 expression in N2a cells overexpressing *miR*-*671*, as determined by western blotting. Control, solvent control; NC, transfection reagent control; control + PbAc, PbAc treatment; mimic + PbAc, PbAc treatment after *miR*-*671* overexpression; NC + PbAc, transfection reagent control with PbAc treatment. **h** Quantitative analysis of results shown in panel (**g**)

To clarify the role of *miR*-*671* in lead-induced neurotoxicity in mice, *miR*-*671* expression was assessed by qRT-PCR. The level of *miR*-*671* was downregulated in the hippocampus and cerebral cortex (Fig. 5a) as well as in N2a cells treated with PbAc (Fig. 5b). Apoptosis in N2a cells overexpressing *miR*-*671* with or without PbAc treatment was assessed by FCM (Fig. 5c, d), TUNEL staining (Fig. 5e, f), and western blot analysis of caspase9 and caspase3 expression (Fig. 5g, h). *MiR*-*671* overexpression inhibited apoptosis according the results of all of the assays. These results indicate that *lncRpa* and *circRar1* promote apoptosis via regulation of *miR*-*671*.

### *MiR*-*671* regulates apoptosis-associated factors

**Fig. 6:**
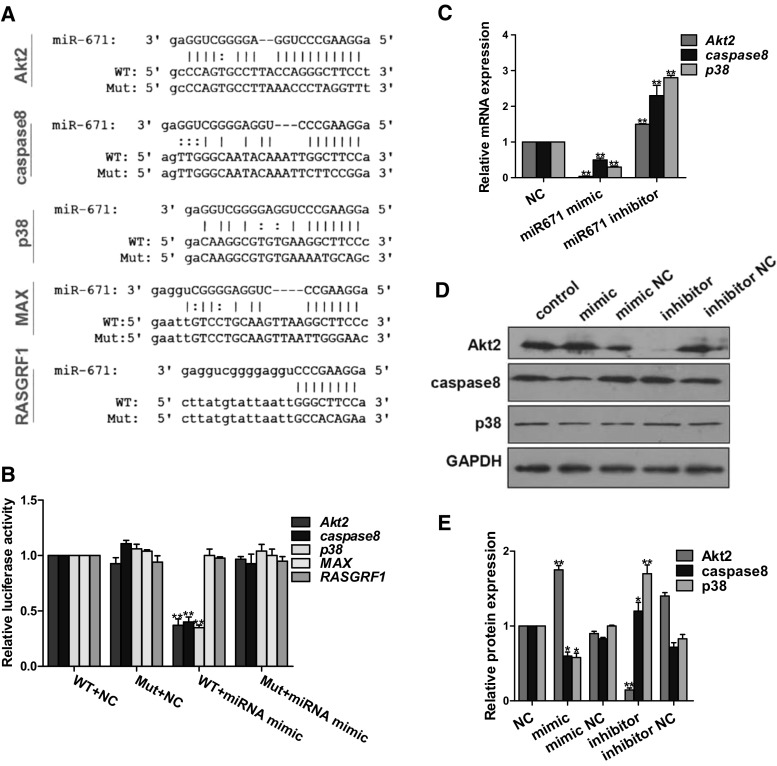
*MiR*-*671* target mRNAs and proteins. **a** Alignment of *miR*-*671* and seed sequences of *Akt2*, *caspase 8*, *p38*, *myc*-*associated factor* (*MA*)*X,* and *Ras protein*-*specific guanine nucleotide releasing factor* (*RASGRF*)*1*. WT, wild-type sequences; Mut, sequence mutated in the dual luciferase reporter gene assay. **b** Luciferase reporter gene assay. WT, wild-type vector; Mut, mutated vector; NC, blank control; miRNA mimic, miRNA overexpression. **c** MRNA expression of *Akt2, caspase 8*, and *p38*. NC, transfection reagent negative control; mimic, *miR*-*671* overexpression; inhibitor, *miR*-*671* knockdown. **d** Expression of Akt2, caspase 8, and p38 protein. Control, blank control; mimic, *miR*-*671* overexpression; mimic NC, control for *miR*-*671* overexpression; inhibitor, *miR*-*671* knockdown; inhibitor NC, control for *miR*-*671* knockdown. **e** Quantitative analysis of results shown in panel (**d**)

Target mRNAs of *miR*-*671* were predicted using RegRNA software. Five apoptosis-associated genes including *Akt2,caspase8, p38, Myc*-*associated factor X (MAX),* and *Ras protein*-*specific guanine nucleotide releasing factor 1 (RASGRF1)* were identified (Fig. 6a). The dual luciferase reporter gene assay was used to determine whether there was a direct interaction between *miR*-*671* and each target (Fig. 6b). *MiR*-*671* overexpression and inhibition in N2a cells resulted in the down- and upregulation of *Akt2, caspase8* and *p38* levels, respectively (Fig. 6c). We also found that the expression of caspase8 and p38 protein was inversely proportional and that of Akt2 was directly proportion to *miR*-*671* expression (Fig. 6d, e). Previous studies have demonstrated the apoptosis-inhibiting function of *miR*-*671*; *Akt2* is presumed to inhibit while *caspase8* and *p38* stimulate apoptosis. Thus, *miR*-*671* inhibits neuronal apoptosis via regulation of apoptosis-associated factors. The discrepancy between *Akt2* mRNA and protein expression may be due to post-transcriptional regulation by factors other than *miR*-*671* (Kim et al. 2007).

### *LncRpa* and *circRar1* regulate apoptosis-associated factors in lead-induced neurotoxicity

**Fig. 7:**
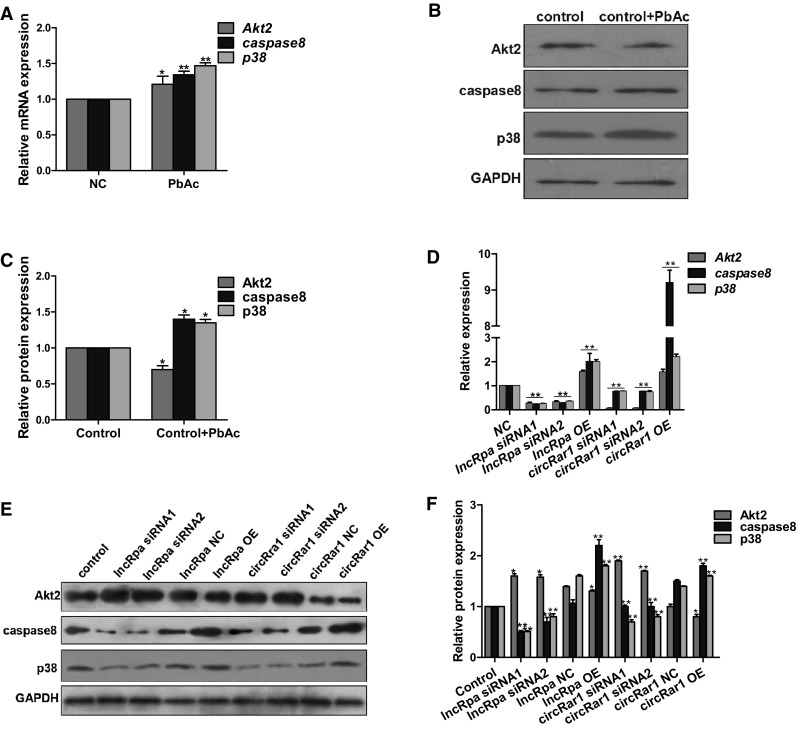
Apoptotic proteins regulated by *lncRpa* and *circRar1* in lead-induced neurotoxicity. **a** Expression of *Akt2, caspase 8*, and *p38* mRNA, as determined by qRT-PCR. NC, cells not treated with PbAc. **b** Expression of Akt2, caspase 8, and p38 protein, as determined by western blotting. NC, cells not treated with PbAc. **c** Quantitative analysis of results shown in panel (**b**). **d** Changes in *Akt2, caspase 8*, and *p38* mRNA expression after knockdown and overexpression of *lncRpa* and *circRar1*, as determined by qRT-PCR. NC, transfection reagent negative control. **e** Akt2, caspase 8, and p38 protein expression after knockdown and overexpression of *lncRpa* and *circRar1*, as determined by western blotting. Control and NC represent transfection reagent and empty vector negative control groups, respectively. **f** Quantitative analysis of results shown in panel (**e**)

To confirm the mechanistic basis for the pro-apoptotic function of *lncRpa* and *circRar1* in lead-induced neurotoxicity, the mRNA levels of *Akt2*, *caspase8*, and *p38* were evaluated in N2a cells exposed to PbAc (Fig. 7a). The transcript levels of all three genes were increased by PbAc treatment, whereas the protein expression of caspase8 and p38 was increased and that of Akt2 was decreased under these conditions (Fig. 7b, c). To investigate the relationship between *lncRpa* and *circRar1* and apoptotic proteins, we knocked down or overexpressed *lncRpa* and *circRar1*. The mRNA levels of *Akt2, caspase8,* and *p38* were decreased by *lncRpa or circRar1* knockdown, whereas their overexpression increased the transcript levels of the three targets (Fig. 7d). The protein levels of caspase8 and p38 were decreased, whereas that of Akt2 was increased by loss of *lncRpa* or *circRar1* (Fig. 7e, f); the opposite trends were observed upon *lncRpa* or *circRar1* overexpression. These data suggest that joint targeting of *miR*-*671* by *lncRpa* and *circRar1* is not the primary reason for the discrepancy between *Akt2* mRNA and protein expression. Moreover, our findings indicate that *lncRpa* and *circRar1* target *caspase8* and *p38* via *miR*-*671* to induce neuronal apoptosis upon lead toxicity.

## Discussion

High-throughput sequencing technology has broadened our understanding of gene regulatory networks. Whole genome sequencing has revealed that about 93 % of the genome is transcribed as RNA, but only 2 % encode proteins (Birney et al. 2007). Although the total number of nucleotides in the human genome is 30 times that of the nematode genome, the number of protein-coding sequences is comparable, which highlights the importance of ncRNA sequences in the regulation of eukaryotic gene expression (Costa 2008).

Environmental toxins such as lead can adversely affect human health, but the molecular mechanisms of lead-induced neurotoxicity are not well understood. Most research in this area has focused on the role of mRNAs (Soliman et al. 2015; Gao et al. 2016) or miRNAs (Li et al. 2015; Martinez-Pacheco et al. 2014), and there is little, if any information on how lncRNAs and circRNAs are involved in lead-induced neurotoxicity. The roles of ncRNAs have been extensively investigated in the context of carcinogenesis and cancer development (Cheng et al. 2015). For example, *H19* is aberrantly expressed in many types of cancer such as liver and bladder cancers and pancreatic ductal carcinoma (Ma et al. 2014; Luo et al. 2013; Tsang and Kwok 2007), while *HOTAIR* has been implicated in various aspects of cancer development (Wu et al. 2014). Genome-wide association studies have shown that most cancer risk loci are found in non-coding sequences (Cheetham et al. 2013). Less is known about circRNAs, despite their prevalence in mammalian cells. These molecules also regulate gene expression at the post-transcriptional level (Memczak et al. 2013), and some have been found to be associated with tumors (Hansen et al. 2013b; Peng et al. 2015; Zhao and Shen 2015).

NcRNAs interact in a complex regulatory network (Supplementary Fig. 1). LncRNAs alter chromatin structure via activation and transport of relevant proteins (Zhao et al. 2008; Tsai et al. 2010; Yao et al. 2010) and are also involved in the regulation of transcription factors (Hung et al. 2011) and mRNA and protein expression (Gong and Maquat 2011; Yoon et al. 2012). In many instances, lncRNAs carry out their functions by modulating the expression of miRNAs at the level of transcription, post-transcription, or splicing (Poliseno et al. 2010; Augoff et al. 2012; Steck et al. 2012; Wang et al. 2010). MiRNAs play a critical role in this regulatory network by directly targeting mRNAs (Orom et al. 2008). Previous studies have shown that miRNAs can be adsorbed by circRNAs, which act as endogenous miRNA competitors (Hansen et al. 2013a, b; Xu et al. 2015).

In this study, we found that lncRNA *lncRpa* and circRNA *circRar1* were differentially expressed in lead-induced neurotoxicity and directly regulated *miR*-*671* expression to promote neuronal apoptosis via upregulation of the pro-apoptotic proteins caspase8 and p38. We also found that *miR*-*671* negatively regulate *circRar1* and *lncRpa*. Thus, *lncRpa* and *circRar1* jointly target *miR*-*671* to modulate the expression of apoptosis-associated proteins in lead-induced neuronal apoptosis. These findings highlight a new mechanism of lead-induced neurotoxicity and provide a insight for the future investigations of the pathological process.

## Electronic supplementary material

Below is the link to the electronic supplementary material.

Supplementary material 1 (DOC 207 kb)

## References

[CR1] Augoff K, McCue B, Plow EF, Sossey-Alaoui K (2012). MiR-31 and its host gene lncRNA LOC554202 are regulated by promoter hypermethylation in triple-negative breast cancer. Mol Cancer.

[CR2] Birney E, Stamatoyannopoulos JA, Dutta A, Guigo R, Gingeras TR, Margulies EH, Weng Z, Snyder M, Dermitzakis ET, Thurman RE (2007). Identification and analysis of functional elements in 1 % of the human genome by the ENCODE pilot project. Nature.

[CR3] Cheetham SW, Gruhl F, Mattick JS, Dinger ME (2013). Long noncoding RNAs and the genetics of cancer. Br J Cancer.

[CR4] Cheng CJ, Bahal R, Babar IA, Pincus Z, Barrera F, Liu C, Svoronos A, Braddock DT, Glazer PM, Engelman DM (2015). MicroRNA silencing for cancer therapy targeted to the tumour microenvironment. Nature.

[CR5] Costa FF (2008). Non-coding RNAs, epigenetics and complexity. Gene.

[CR6] Gao H, Liu CP, Song SQ, Fu J (2016) Effects of dietary selenium against lead toxicity on mRNA levels of 25 selenoprotein genes in the cartilage tissue of broiler chicken. Biol Trace Elem Res 172(1):234–241. doi:10.1007/s12011-015-0579-x 10.1007/s12011-015-0579-x 26643179

[CR7] Gong C, Maquat LE (2011). LncRNAs transactivate STAU1-mediated mRNA decay by duplexing with 3′ UTRs via Alu elements. Nature.

[CR8] Hansen TB, Jensen TI, Clausen BH, Bramsen JB, Finsen B, Damgaard CK, Kjems J (2013). Natural RNA circles function as efficient microRNA sponges. Nature.

[CR9] Hansen TB, Kjems J, Damgaard CK (2013). Circular RNA and miR-7 in cancer. Cancer Res.

[CR10] Hsu PW, Lin LZ, Hsu SD, Hsu JB, Huang HD (2007). ViTa: prediction of host microRNAs targets on viruses. Nucleic Acids Res.

[CR11] Huang HY, Chien CH, Jen KH, Huang HD (2006). RegRNA: an integrated web server for identifying regulatory RNA motifs and elements. Nucleic Acids Res.

[CR12] Hung T, Wang Y, Lin MF, Koegel AK, Kotake Y, Grant GD, Horlings HM, Shah N, Umbricht C, Wang P (2011). Extensive and coordinated transcription of noncoding RNAs within cell-cycle promoters. Nat Genet.

[CR13] Kim R, Emi M, Tanabe K (2007). Cancer immunoediting from immune surveillance to immune escape. Immunology.

[CR14] Lasda E, Parker R (2014). Circular RNAs: diversity of form and function. RNA.

[CR15] Li Q, Kappil MA, Li A, Dassanayake PS, Darrah TH, Friedman AE, Friedman M, Lambertini L, Landrigan P, Stodgell CJ (2015). Exploring the associations between microRNA expression profiles and environmental pollutants in human placenta from the National Children’s Study (NCS). Epigenetics US.

[CR16] Luo M, Li Z, Wang W, Zeng Y, Liu Z, Qiu J (2013). Upregulated H19 contributes to bladder cancer cell proliferation by regulating ID2 expression. FEBS J.

[CR17] Ma C, Nong K, Zhu H, Wang W, Huang X, Yuan Z, Ai K (2014). H19 promotes pancreatic cancer metastasis by derepressing let-7′s suppression on its target HMGA2-mediated EMT. Tumour Biol.

[CR18] Martinez-Pacheco M, Hidalgo-Miranda A, Romero-Cordoba S, Valverde M, Rojas E (2014). MRNA and miRNA expression patterns associated to pathways linked to metal mixture health effects. Gene.

[CR19] Maruyama R, Suzuki H (2012). Long noncoding RNA involvement in cancer. BMB Rep.

[CR20] Memczak S, Jens M, Elefsinioti A, Torti F, Krueger J, Rybak A, Maier L, Mackowiak SD, Gregersen LH, Munschauer M (2013). Circular RNAs are a large class of animal RNAs with regulatory potency. Nature.

[CR21] Mercer TR, Dinger ME, Mattick JS (2009). Long non-coding RNAs: insights into functions. Nat Rev Genet.

[CR22] Nan A, Zhou X, Chen L, Liu M, Zhang N, Zhang L, Luo Y, Liu Z, Dai L, Jiang Y (2016). A transcribed ultraconserved noncoding RNA, Uc.173, is a key molecule for the inhibition of lead-induced neuronal apoptosis. Oncotarget.

[CR23] Okazaki Y, Furuno M, Kasukawa T, Adachi J, Bono H, Kondo S, Nikaido I, Osato N, Saito R, Suzuki H (2002). Analysis of the mouse transcriptome based on functional annotation of 60,770 full-length cDNAs. Nature.

[CR24] Orom UA, Nielsen FC, Lund AH (2008). MicroRNA-10a binds the 5′UTR of ribosomal protein mRNAs and enhances their translation. Mol Cell.

[CR25] Padmashree D, Swamy NR (2015). Computational identification of putative miRNAs and their target genes in pathogenic amoeba Naegleria fowleri. Bioinformation.

[CR26] Peng L, Yuan XQ, Li GC (2015). The emerging landscape of circular RNA ciRS-7 in cancer (Review). Oncol Rep.

[CR27] Poliseno L, Salmena L, Zhang J, Carver B, Haveman WJ, Pandolfi PP (2010). A coding-independent function of gene and pseudogene mRNAs regulates tumour biology. Nature.

[CR28] Soliman MM, Baiomy AA, Yassin MH (2015). Molecular and histopathological study on the ameliorative effects of curcumin against lead acetate-Induced hepatotoxicity and nephrototoxicity in wistar rats. Biol Trace Elem Res.

[CR29] Steck E, Boeuf S, Gabler J, Werth N, Schnatzer P, Diederichs S, Richter W (2012). Regulation of H19 and its encoded microRNA-675 in osteoarthritis and under anabolic and catabolic in vitro conditions. J Mol Med (Berl).

[CR30] Storz G (2002). An expanding universe of noncoding RNAs. Science.

[CR31] Taft RJ, Pang KC, Mercer TR, Dinger M, Mattick JS (2010). Non-coding RNAs: regulators of disease. J Pathol.

[CR32] Tsai MC, Manor O, Wan Y, Mosammaparast N, Wang JK, Lan F, Shi Y, Segal E, Chang HY (2010). Long noncoding RNA as modular scaffold of histone modification complexes. Science.

[CR33] Tsang WP, Kwok TT (2007). Riboregulator H19 induction of MDR1-associated drug resistance in human hepatocellular carcinoma cells. Oncogene.

[CR34] Wang HL, Chen XT, Yin ST, Liu J, Tang ML, Wu CY, Ruan DY (2008). Opposite effects of alpha-lipoic acid on antioxidation and long-term potentiation in control and chronically lead-exposed rats. Naunyn Schm Arch Pharmacol.

[CR35] Wang J, Liu X, Wu H, Ni P, Gu Z, Qiao Y, Chen N, Sun F, Fan Q (2010). CREB up-regulates long non-coding RNA, HULC expression through interaction with microRNA-372 in liver cancer. Nucleic Acids Res.

[CR36] Wilusz JE, Sharp PA (2013). A circuitous route to noncoding RNA. Science.

[CR37] Wu Y, Zhang L, Wang Y, Li H, Ren X, Wei F, Yu W, Wang X, Zhang L, Yu J, Hao X (2014). Long noncoding RNA HOTAIR involvement in cancer. Tumour Biol.

[CR38] Xu H, Guo S, Li YuP (2015). The circular RNA Cdr1as, via miR-7 and its targets, regulates insulin transcription and secretion in islet cells. Sci Rep.

[CR39] Yao H, Brick K, Evrard Y, Xiao T, Camerini-Otero RD, Felsenfeld G (2010). Mediation of CTCF transcriptional insulation by DEAD-box RNA-binding protein p68 and steroid receptor RNA activator SRA. Genes Dev.

[CR40] Yoon JH, Abdelmohsen K, Srikantan S, Yang X, Martindale JL, De S, Huarte M, Zhan M, Becker KG, Gorospe M (2012). LincRNA-p21 suppresses target mRNA translation. Mol Cell.

[CR41] Zhang Y, Zhang XO, Chen T, Xiang JF, Yin QF, Xing YH, Zhu S, Yang L, Chen LL (2013). Circular intronic long noncoding RNAs. Mol Cell.

[CR42] Zhang XO, Wang HB, Zhang Y, Lu X, Chen LL, Yang L (2014). Complementary sequence-mediated exon circularization. Cell.

[CR43] Zhao ZJ, Shen J (2015) Circular RNA participates in the carcinogenesis and the malignant behavior of cancer. RNA Biol 9:1–8. doi:10.1080/15476286.2015.1122162 10.1080/15476286.2015.1122162 PMC5449088 26649774

[CR44] Zhao J, Sun BK, Erwin JA, Song JJ, Lee JT (2008). Polycomb proteins targeted by a short repeat RNA to the mouse X chromosome. Science.

