# Supplementary material for: A novel regulatory network among LncRpa, CircRar1, MiR-671 and apoptotic genes promotes lead-induced neuronal cell apoptosis
Source: Arch Toxicol. 2016 Sep 7;91(4):1671–84. doi: 10.1007/s00204-016-1837-1 (PMC5364257; doi:10.1007/s00204-016-1837-1)
Supplement: Supplementary file 1 — Supplementary material 1 (DOC 207 kb) [file 204_2016_1837_MOESM1_ESM.doc]

**Supplementary Table 1**

Primer sequences for siRNAs against *lncRpa* and *circRar1* and for overexpression vectors

| **Type** | **Target gene** | | **Primer** | **Sequence** | |  |  |  |  |
| --- | --- | --- | --- | --- | --- | --- | --- | --- | --- |
| siRNA | *lncRpa* |  | 1F | GUCAGACAAUGAGGGUUGU UU | | | |  |  |
|  |  |  | 1R | ACAACCCUCAUUGUCUGAC UU | | | |  |  |
|  |  |  | 2F | GGACCUGCAAGUCAGACAA UU | | | |  |  |
|  |  |  | 2R | UUGUCUGACUUGCAGGUCC UU | | | |  |  |
|  |  |  | 3F | GGCAUGAAUAGUCCUUUCU UU | | | |  |  |
|  |  |  | 3R | AGAAAGGACUAUUCAUGCC UU | | | |  |  |
|  | *circRar1* |  | 1F | CUCUGCCUCUGCAGGGUAA UU | | | |  |  |
|  |  |  | 1R | UUACCCUGCAGAGGCAGAG UU | | | |  |  |
|  |  |  | 2F | CACUCUGCCUCUGCAGGGU UU | | | |  |  |
|  |  |  | 2R | ACCCUGCAGAGGCAGAGUG UU | | | |  |  |
|  |  |  | 3F | CAGGCAUUGUACUUCACCU UU | | | |  |  |
|  |  |  | 3R | AGGUGAAGUACAAUGCCUG UU | | | |  |  |
| OE | *lncRpa* |  | F | CTAGCGTTTAAACTTAAGTCAGACCAGAGATCTGGAA | | | | | |
|  |  |  | R | ACGGGCCCTCTAGACCCTCATACTCTTTGGAAAAAT | | | | | |
|  | *circRar1* |  | F | AGTATGGCACGCCAGAGTTT | | |  |  |  |
|  |  |  | R | AGCCAGGGCTACACAGAGAA | | |  |  |  |
|  |  |  |  |  |  |  |  |  |  |

**Supplementary Table 2**

Sequences of probes used in the FISH experiment

| **FISH probe** |  |  |  |
| --- | --- | --- | --- |
| *lncRpa* | GATCTAGCACTCACCATGTCTCA | | |
| *circRar1* | GGGTCCTTACCCTGCAGAGGCA | | |
| *miR-671* | CTCCAGCCCCTCCAGGGCTTCCT | | |

**Supplementary Table 3**

Primer sequences used for qRT-PCR

| **type** | **Target gene** | | **Primer** | **siRNA Sequence** |  |
| --- | --- | --- | --- | --- | --- |
| qPCR | *U6* |  | F | CGCTTCGGCAGCACATATAC | |
|  |  |  | R | AAATATGGAACGCTTCACGA | |
|  | *GAPDH* |  | F | TGTGTCCGTCGTGGATCTGA | |
|  |  |  | R | TTGCTGTTGAAGTCGCAGGAG | |
|  | *lncRpa* |  | F | TGCTATTGGGTGCTGTGAGG | |
|  |  |  | R | TAGGAAAAATGTCACACACCTGC | |
|  | *circRar1* |  | F | AGATTTGCCTCTGGTGTCCC | |
|  |  |  | R | GACTGGTGCATGTGAAACCC | |
|  | *miR-671* |  | F | AGGAAGCCCUGGAGGGGCUGGAG | |
|  | *miR-218* |  | F | UUGUGCUUGAUCUAACCAUGU | |
|  | *caspase8* |  | F | CAGACAGAGAAGGGGCTTGG | |
|  |  |  | R | CATCCTCGATGGTCTCCTGC | |
|  | *Akt2* |  | F | TGGCTGGAAAAGGCGGTATT | |
|  |  |  | R | GCTCGTTCCCGCTCCTTATT | |
|  | *p38* |  | F | TGGATATTTGGTCCGTGGGC | |
|  |  |  | R | TTCTTGCCTCATGGCTTGGC | |
|  | *MAX* |  | F | GGCTTGTTGTTGTCGGTGAC | |
|  |  |  | R | CTTGTCAGCGTCGCTCTCC | |
|  | *RASGRF1* |  | F | AAAGCCATCCGGCTTAACGA | |
|  |  |  | R | CGTCGACTCCTGCTATGACG | |

**Supplementary Table 4**

Sequences of probes used in the RAP experiment

| RAP probe | |  |  |  |  |  |  |
| --- | --- | --- | --- | --- | --- | --- | --- |
| *lncRpa* |  | CACACTTTAAACTAGAGGAGGGGCTTGAATGTTTCTCCGTGTCTT | | | | |  |
|  |  | TAAGCTCCGTTTTTGAAACATGATCTTCATCCACATCTTGAAGTT | | | | |  |
| *circRar1* |  | GTCTGACCCGGGACTGCAAGCTCTATGAATTTTTCCAAAGAGTATGATTT | | | | | |

**Supplementary Figure 1**

ncRNAs regulatory network.

**
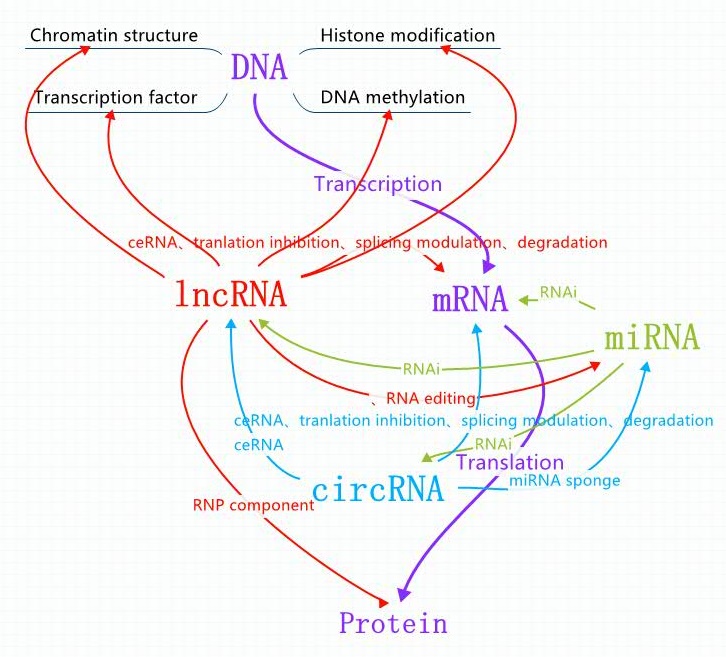
**
